# Life-Cycle Assessment and Acoustic Simulation of Drywall Building Partitions with Bio-Based Materials

**DOI:** 10.3390/polym12091965

**Published:** 2020-08-30

**Authors:** Alberto Quintana-Gallardo, Jesús Alba, Romina del Rey, José E. Crespo-Amorós, Ignacio Guillén-Guillamón

**Affiliations:** 1 Center for Physics Technologies (CTFAMA), Universitat Politècnica de València, 46022 València, Spain; iguillen@fis.upv.es; 2 Center for Physics Technologies (CTFAMA), Escola Politècnica Superior de Gandia, Universitat Politècnica de València, 46730 Gandia, Spain; jesalba@fis.upv.es (J.A.); roderey@fis.upv.es (R.d.R.); 3 Instituto de Tecnología de Materiales, Escuela Politécnica Superior de Alcoy, Universitat Politècnica de València, 03801 Alcoy, Spain; jocream@dimm.upv.es

**Keywords:** life cycle assessment, sustainable building materials, bio-epoxy composites, building acoustics

## Abstract

The ecological transition is a process the building industry is bound to undertake. This study aimed to develop new bio-based building partition typologies and to determine if they are suitable ecological alternatives to the conventional non-renewable ones used today. This work started with the development of a bio-based epoxy composite board and a waste-based sheep wool acoustic absorbent. Six different partition typologies combining conventional and bio-based materials were analyzed. A drywall partition composed of gypsum plasterboard and mineral wool was used as the baseline. First, a cradle-to-gate life cycle assessment was performed to compare their environmental impacts. Secondly, a mathematical simulation was performed to evaluate their airborne acoustic insulation. The LCA results show a 50% decrease in the amount of CO_2_ equivalent emitted when replacing plasterboard with bio-composite boards. The bio-composites lower the overall environmental impact by 40%. In the case of the acoustic absorbents, replacing the mineral wool with cellulose or sheep wool decreases the carbon emissions and the overall environmental impact of the partition from 4% and 6%, respectively. However, while the bio-based acoustic absorbents used offer good acoustic results, the bio-composites have a lower airborne acoustic insulation than conventional gypsum plasterboard.

## 1. Introduction

The building industry is responsible for one-third of the total carbon emissions in the world. Building materials, which usually come from non-renewable sources, are responsible for a big part of those emissions [1]. Transitioning towards sustainable constructions requires finding materials with a lower impact over the environment, and using bio-based materials is possibly the best route [2]. However, this transition is an especially complex process for the building industry [3]. Construction materials need to have a specific set of characteristics to ensure proper habitability for the dwellers. Potential customers need the assurance that the materials used will perform adequately for decades before investing in a new home. This situation makes stakeholders lean towards safer choices, which tend to have higher impacts on the environment. There is only one solution to counteract this tendency, to prove that the new bio-based construction materials are not only better for the environment but can also perform to the required standards. The life cycle assessment (LCA) methodology has a proven record of success in the evaluation of the environmental performance of both building and building materials [4]. Over the last decade, the number of studies dealing with the LCA of sustainable building materials has grown substantially [5]. Through this methodology, it is possible to assess the environmental footprint of any material and to establish a comparison with other materials in a reliable way. Having reliable information about the effect that a particular material has over the environment is crucial to foster the use of new sustainable alternatives.

Besides the environmental performance, building requirements greatly vary depending on the building element. Whereas the building envelope must provide sufficient thermal and acoustic insulation, partition systems only need to be able to perform acoustically. Despite being sometimes forgotten, acoustic insulation is one of the key aspects to consider in terms of habitability. Having properly insulated walls has a major impact on comfort [6]. One of the most widely spread partition typologies is drywall. Drywall is typically formed by a frame, two plasterboards on each side of the frame, and an inner acoustic absorber. Plasterboard is manufactured from gypsum rocks, which are non-renewable minerals extracted from quarries. The manufacturing process, as it is discussed in subsequent sections, involves using a high amount of energy. The inner layer, which provides acoustic absorption, can be composed of many different materials, the most common being mineral wool. The primary raw material to produce mineral wool is basalt. Besides the impacts related to the extraction of minerals, basalt is heated to its melting point during the production process of mineral wool, which requires a large amount of energy. The main goal of this article is to explore the possibilities of using bio-based alternatives to plasterboard in the building market.

## 2. Materials and Methods

**Figure 1:**
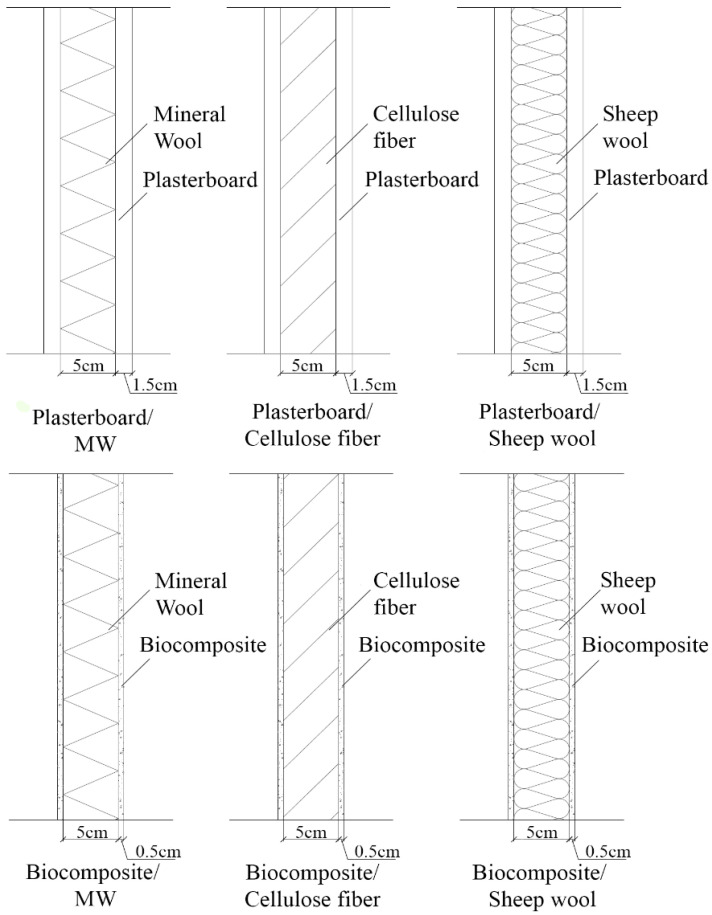
Partition typologies under study.

The environmental impacts and the airborne acoustic insulation of several building partitions combining conventional and new bio-based materials are studied. Conventional drywall containing plasterboard and mineral wool is used as the baseline. A bio-epoxy composite board with flax fiber as its solid filling is proposed as an alternative to plasterboard. The name of this bio-epoxy resin is Supersap, produced by the company Entropy Resins. The company claims to reduce CO_2_ emissions by 50% compared to regular epoxy resins [7]. Flax was chosen as the solid filling because of its regional availability and its mechanical properties [8]. A comparison between gypsum plasterboard and Supersap composites has been previously performed [9]. However, neither the influence those bio-composites have over the total environmental impacts of the partition nor their acoustic performance have been assessed. Two different bio-based materials are considered to replace mineral wool as the acoustic absorbent. The first one is recycled cellulose, which is gaining popularity as a sustainable acoustic absorbent and thermal insulator [10]. The acoustic properties of cellulose have been previously studied [11]. However, the relation between its acoustic properties and its environmental impacts has not been assessed yet. The second alternative is produced using the wool waste generated during the manufacturing process of sheep wool and adding PET in its formulation to increase its rigidity. This material was developed by researchers at the Polytechnic University of Valencia in conjunction with a sheep wool manufacturing plant. The acoustic absorption of this material has been previously studied, but an assessment of its environmental impacts has not been performed yet [12]. When it comes to the frame of drywall partitions, it can be built using many kinds of materials, wood being the most widely spread in many parts of the world. However, in countries such as Spain, drywall frames are usually made of galvanized steel, probably due to resource availability. For that reason, galvanized steel is used as the material for the frame in this work. The using wood as the frame of drywall partitions is outside the scope of this study. The different configurations analyzed in this study are represented in Figure 1. The main components of each typology are described in Table 1. The proportions of Supersap bio-epoxy resin and flax fiber are specified in Table 2. The physical characteristics of the bio-composite board were tested in the university facilities, Table 3.

### 2.1. Acoustic Simulation Methodology

**Figure 2:**
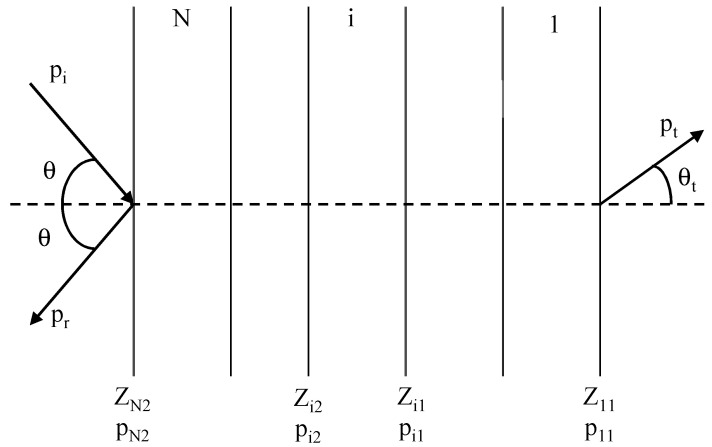
Ookura & Saito simulation process. p_t_: transmitted pressure, p_i_: incident pressure, p_r_: reflected pressure, θ: angle of incidence of the acoustic wave, Z: complex characteristic impedance. N references the number of elements. The physical parameters are numbered with the subscript i = 1, 2, …, n to indicate the element and a second subscript to indicate the right or the left face of the element.

The airborne sound insulation of each solution has been computed using the simulation software Aisla 3 [13]. This software applies the mathematical model proposed by Ookura & Saito [14] and Chen & Jan [15], which uses the mechanical data of the materials to determine the coupling impedance (Z_ij_) between layers. The simulation process is summarized in Figure 2.

The critical frequency (*f_c_*) is obtained using the expression Equation (1) where *D* (N·m) is the flexion stiffness of the board, *c* the speed of sound, η the loss factor of the material, and m the mass per surface unit of the material.

(1) $$f_{c}=\frac{c^{2}}{2\pi }\sqrt{\frac{m}{D}}$$

The cellulose fiber absorbent formulas are obtained from [11]. The formulas to assess sheep wool behavior can be found in [12]. The data used for the calculations, which were obtained by testing the materials in the university facilities, can be seen in Table 4. The margin of error due to the material values was assessed [16], concluding that there is a possible deviation of 2 to 3 decibels. This margin of error is accepted by the Spanish technical building code [17]. The transmission coefficient is assessed from the simulation results according to the ISO standard 717-1 [18].

### 2.2. Life Cycle Assessment Methodology

A cradle-to-gate life cycle assessment (LCA) of the different partition configurations has been carried out. The LCA follows the framework of the ISO 14040 [22].

#### 2.2.1. Functional Unit

The functional unit considered for the study is 1 m^2^. This is the most adequate functional unit for evaluating the environmental impacts of interior partitions. Due to the different thicknesses of each configuration and the fact that they are multi-layered elements, using mass or volume reference units would not be suitable for this study.

#### 2.2.2. Inventory Analysis

An inventory analysis has been performed according to the ISO 14044 [23]. The inventory analysis, which accounts for any activity susceptible to having an impact over the environment, has been carried out using Simapro 9.0.0.35, the last version of one of the most popular software programs used for LCA calculations. All the information used for this study combines data provided by the industry, data collected from the laboratory, and data extracted from databases. The production company of the sheep wool acoustic absorber supplied the necessary data related to their product. The information about the bio-composite assembly was obtained during the testing and manufacturing of the boards in the laboratory. In the case of the production of the Supersap bio-epoxy resin, the information came directly from the manufacturer. The rest of the data comes from the Ecoinvent V3.5 database [24]. Ecoinvent is a not-for-profit organization founded by institutes of the ETH Domain and the Swiss Federal Offices. This database collects highly reliable information due to its peer review process [25]. The production processes of each material used in this study are summarized as follows:

Production process of gypsum plasterboard: Gypsum plasterboards are composed of a plaster core with one protective cellulose layer on each side. The manufacturing process begins by extracting the gypsum rocks from the quarry. Those rocks, which have a maximum diameter of 5 cm, are taken to a factory. Once the rocks get there, they are ground into a fine powder and put into an oven at 160 °C. This process turns the original material into stucco. The stucco is then mixed with water creating slurry. This slurry is poured onto a cellulose layer, and then another cellulose layer is unrolled on top of it. After a compacting process in which the core reaches the desired thickness, it hardens and is prepared to be cut at the intended size. As a final step, the boards are put in an oven to remove any remaining moisture.

Production process of the bio-epoxy composite boards: As mentioned in previous sections, the bio-composite boards are made of a bio-epoxy matrix and a flax fiber solid filling. The obtention of flax fibers begins with the harvest. The harvested fibers are submitted to a retting process intended to dissolve most of the cellular tissues and pectin that surround the fiber. Subsequently, the flax fibers go through scutching, which involves crushing the stems with a pair of fluted rollers and beating them with a rotating blade, therefore making the inner body tissue fall off. The scutched fibers are then hackled to remove the remaining impurities and wood particles, obtaining slivers and hackled tows. The last step is spinning. Although there are several kinds of spinning processes, the one used for these kinds of applications is usually dry spinning. Dry spinning produces coarser fibers than wet spinning but uses fewer chemical products. The bio-epoxy resin used is manufactured by the company Entropy Resins. The feature that makes this epoxy resin different from others is the biological content in its formulation. The epoxy class analyzed in this study is phenolic glycidyl ether. This kind of epoxy resin is obtained by the reaction of epichlorohydrin (ECH), a key component of the vast majority of commercial epoxy resins, and a phenol group, in this case bisphenol-A (BPA) which is the most widely used today [26]. However, the manufacturing process of Supersap has some key differences compared to the conventional epoxy resins. The specifics about those differences are subject to a confidentiality agreement. The last step of the bio-composite manufacturing is the assembly of the matrix and the filler. In this case, the method used was resin infusion. The quantity of flax fiber required is placed inside a mold. Then, the resin is introduced through tubes by vacuum suction. This method is one of the most modern methods of composite manufacturing [27]. The process described was conducted in the laboratory of the Polytechnic University of Valencia.

Production process of mineral wool: The production process of mineral wool starts with the extraction of basalt from the quarry and its transportation to a manufacturing plant. Once the raw material is in the plant, it is loaded into a cupola in alternating layers with coke. The commonly used proportions are five parts of mineral and one part of coke. Then, the coke is burnt, which heats the basalt to a temperature from 1300 °C to 1650 °C. Once the basalt melts, it exits the cupola and is taken through pipes to a fiberization machine. This machine has a rotor system that revolves at high speed to take advantage of the centrifugal force to distribute the material over its round surface. At the end of this process, the material is discharged from the machine and poured onto a conveyor belt that takes it to a blowchamber. In this chamber, air is blown to the material to create a wool blanket. The wool blanket is then compressed to reach the appropriate density while air is passed through the blanket until the binder is baked. The manufacturing ends after cooling and cutting the material to the desired size [28].

Production process of sheep wool: As explained in previous sections, the development of the sheep wool absorbent has been made in conjunction with sheep wool industry partners as a part of this research project. The sheep wool insulating panels are primarily made from the waste generated in the wool industry. The production process begins by washing, cutting, and drying the wool fibers. After that, the material undergoes a carding process. Then, the wool is mixed with PET fibers in a thermobonding process. The inventory of the material can be seen in Table 5.

Production process of cellulose fiber: The insulating cellulose fiber panels are commonly made out of wastepaper. The process begins with taking the collected paper waste to the factory. Once there, the waste is loaded onto a conveyor belt which takes it to a primary mixer. The primary mixer prepares the waste for shredding by removing any metals and other non-desired elements in the mix and washing it using anhydrous borax. Once that process is completed, the waste is loaded into the shredder where it is reduced to small particles and mixed with boric acid. Once the particles reach the desired size, they are taken to a fiberizer where the particle size is reduced up to 4 mm and mixed with more boric acid [29]. The inventory of the material can be seen in Table 6.

#### 2.2.3. Allocation Principle

As recommended by the ISO standard 14040, allocation has been avoided when it has been possible. However, in some cases, due to the multifunctional nature of some processes and materials, allocation is required. The consequential approach was chosen to overcome the difficulties associated with multifunction processes. This approach uses substitution to resolve multifunctionality in datasets instead of allocation [30].

#### 2.2.4. Evaluation Methods

The evaluation methods chosen for this comparative LCA are the IPCC.GWP 100a and the ReCiPe method. Developed by the intergovernmental panel on climate change [31], the IPCC.GWP method is used to calculate the greenhouse emissions of each typology separately. In the case of the ReCiPe method, created by the Dutch public administration [32], it is used to calculate the environmental impacts divided into several categories. The ReCiPe method uses two different approaches: the midpoint and the endpoint approach. While the midpoint approach keeps each category separate, the endpoint normalizes and weighs them to add them up and offer a single figure representing the environmental impact. Both the midpoint and the endpoint approach were used in this study.

## 3. Results

### 3.1. Airborne Noise Insulation Results

**Figure 3:**
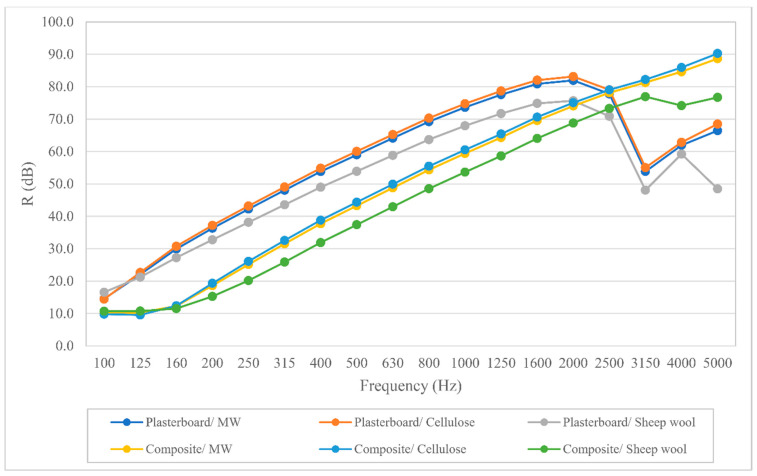
Airborne noise insulation simulation results of each typology under study.

The airborne noise insulation results are depicted in Figure 3. As can be seen, the partitions using the bio-composite boards have lower insulation values in most frequencies than the ones using plasterboard. Therefore, as it is reflected in Table 7, the sound reduction index reached by the partitions using plasterboard is higher than the one obtained by the partitions with bio-composite boards. As it is discussed in subsequent sections, the main reason behind the lower acoustic insulation is likely to be the lower mass of the bio-epoxy composite compared to plasterboard.

### 3.2. Life Cycle Assessment Results

In this section, the environmental impacts obtained by performing an LCA are assessed using the IPCC GWP 100a method and the ReCiPe method.

#### 3.2.1. IPCC GWP 100a Method Comparative Results: Carbon Dioxide Emissions

**Figure 4:**
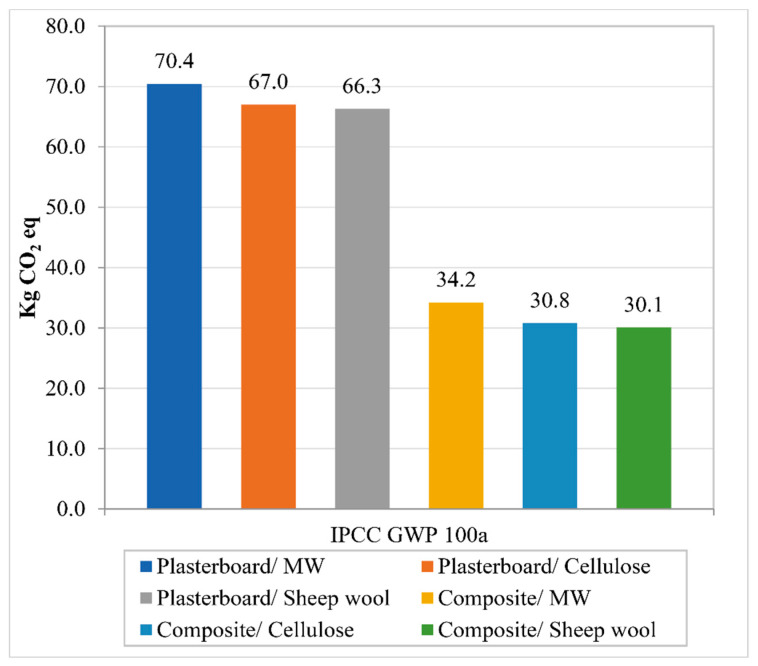
Carbon dioxide equivalent emitted by each typology. IPCC GWP 100a.

The IPCC GWP 100 results, depicted in Figure 4, show a clear gap between the partitions with plasterboard and the ones with bio-composite boards. The CO_2_ emissions over the production of the partitions using bio-composite boards are 50% lower than the ones of the typologies with plasterboard. In the case of the acoustic absorbents, the influence they have over the total carbon emissions is not as substantial. When compared to mineral wool, cellulose fiber reduces carbon emissions by around 3.5% and the sheep wool absorbent by slightly more than 4%. The carbon emissions of each material used are represented in Table 8 to illustrate their contribution to the total carbon emissions of every respective partition typology. As can be seen, plasterboard is the material with the higher CO_2_ emissions. Its contribution to the total carbon emissions of each partition ranges from 82.2% to 87.3%. When it comes to the acoustic absorbents, mineral wool accounts for almost 7% of the total carbon emissions. This percentage is lower in the case of cellulose fiber, 2.17%, and even lower using sheep wool, 1.12%. The percentual impact the bio-composites have is smaller than the one obtained with plasterboard, ranging from 63.3% to 72%. In this case, the percentual impact the acoustic absorbent has is higher. It accounts for 14.3% using mineral wool, 4.73% with cellulose fiber, and 2.48% with sheep wool. The galvanized steel used for the frame is responsible for around 10% of the total carbon emissions, in the case of the partitions using plasterboard, and up to 25% in the partitions with bio-composite boards.

#### 3.2.2. ReCiPe Method Comparative Results

**Figure 5:**
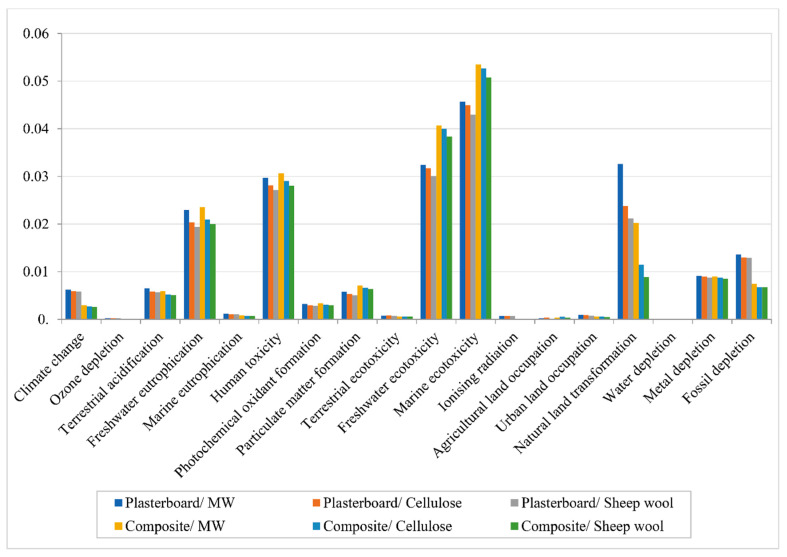
ReCiPe midpoint normalization results.

ReCiPe midpoint: The ReCiPe midpoint method classifies the environmental impacts in eighteen different categories. As can be seen in Table 9, the impact results of the three partitions with plasterboard are higher in almost every category. Only the results in marine ecotoxicity and water depletion are higher in the partitions containing the bio-composites. However, looking at the raw figures can be misleading due to the lack of comparative perspective that they offer. That is the reason why the ReCiPe midpoint offers the possibility to normalize them. The results are displayed in Figure 5. Despite the normalization process, it is still complex to identify which solutions have an overall less impacting production process. While the freshwater and marine ecotoxicity results have smaller impacts on the solutions using gypsum plasterboard, other categories such as climate change, natural land transformation, and fossil depletion show higher impact results. The next step is to use the ReCiPe endpoint method to group the categories.

**Figure 6:**
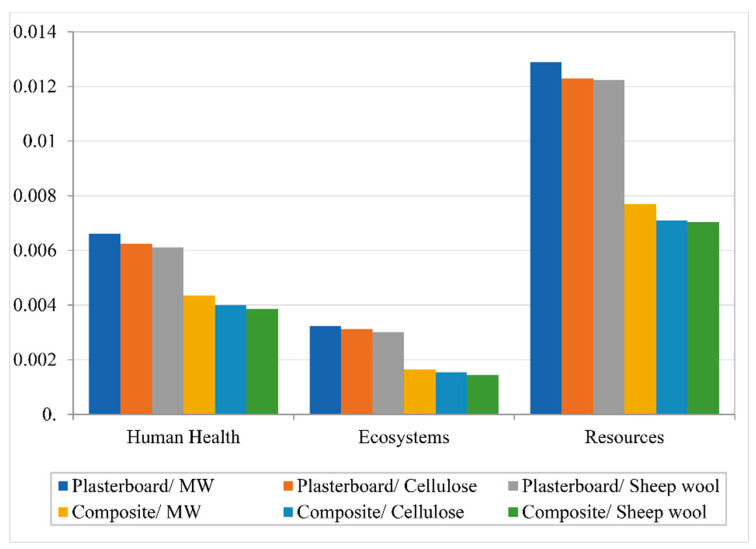
ReCiPe endpoint normalization results.

**Figure 7:**
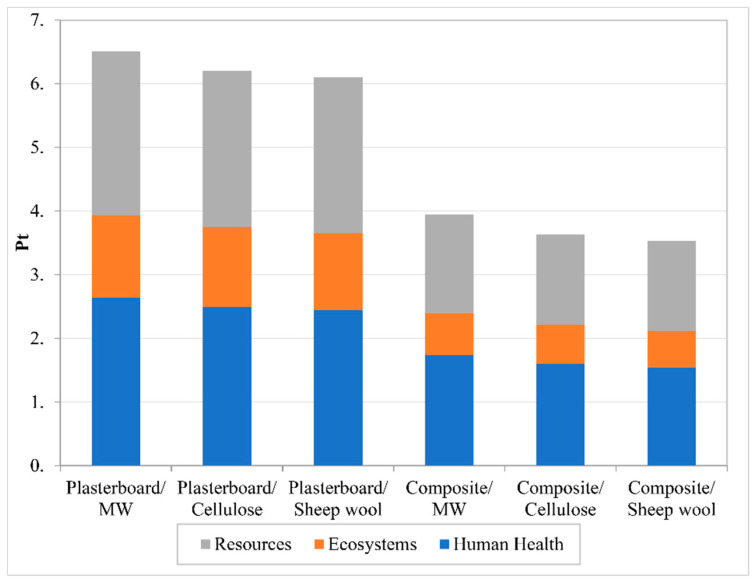
ReCiPe endpoint single-score results.

ReCiPe endpoint: The ReCiPe endpoint methodology is used to simplify the results displayed in the ReCiPe midpoint. After a normalization process, the ReCiPe endpoint groups the results in three different categories, human health, ecosystems, and resources, Figure 6. These categories are the result of normalizing and adding up the categories of the Recipe midpoint method. The results in the three categories show a clear difference between the solutions using plasterboard and the ones using the bio-composite boards. The use of the bio-epoxy composite lowers the environmental impact in all of them, especially in Resources. As for the influence of the inner filling, there is a relatively small reduction in the impacts when mineral wool is replaced by either cellulose fiber or sheep wool. As the final step, the results are weighted and grouped in a single score result, Figure 7. Following the same trend identified in previous results, the substitution of the plasterboard for the bio-epoxy composite reduces the overall environmental impact. The results oscillate from a 30% reduction in case of the one filled with sheep wool to a 40% reduction in the case of the one filled with mineral wool.

## 4. Discussion

The results obtained in this study highlight the potential reduction in the environmental impacts that can be attained by using bio-based materials in building partitions. The life cycle assessment results indicate that plasterboard is the main contributor to the environmental impacts in drywall partitions. The results also show a significant reduction in the environmental impacts generated when plasterboard is replaced with the bio-composite boards. In the case of the carbon emissions, those assessed using the IPCC GWP method show that plasterboard is responsible for more than 80% of the carbon emissions of the partition. This indicates the need to find ecological alternatives to that building material. Results also show that using the proposed bio-epoxy boards reduces carbon emissions by more than 50%. Besides carbon emissions, the categories analyzed using the ReCiPe endpoint method show that the use of the bio-composite boards decreases human health-related impacts by 35%, impacts related to ecosystems by around 50%, and the ones related to resource depletion by approximately 40%. Therefore, it can be stated that the LCA results reflect the environmental benefits of using the bio-composite boards instead of conventional plasterboard. When it comes to the absorbent materials, the results indicate that the influence they have over the total environmental impact of the partition is smaller compared to the boards. Replacing mineral wool by cellulose fiber reduces the carbon dioxide equivalent by 4.7%, and 6.35% in the case of sheep wool. As it was the case with the results obtained in the IPCC GWP 100a method, the ReCiPe endpoint method shows that replacing mineral wool with cellulose reduces the impacts by 4.7% and 6.35% with the sheep wool absorbent. Although those percentages are small when comparing the impacts of the partitions as a whole, the results show that the carbon emissions of the sheep wool absorbent are around 85% lower than the ones generated by mineral wool, and 70% lower in the case of cellulose fiber. Due to the enormous volume of materials that it is used in the building industry, that reduction in the carbon emissions over hundreds of buildings would have a significant effect in combating climate change. One aspect worth mentioning is the impact on the environment of the steel frame of the partitions. The steel frame, the only element that has not been replaced with a bio-based alternative in this study, is responsible for 7.6 kg of CO_2_ equivalent per square meter, which accounts for 10% to 25% of the total carbon emissions of the partition depending on the board used. Drywall frames in countries such as Spain are commonly made of galvanized steel, despite wood being probably the most common material worldwide in this kind of application. The use of steel is possibly due to material availability and the several blast furnaces in Spain during the twentieth century. Depending on the tree species, it would probably be possible to significantly reduce the environmental impacts of the partitions by using wood to build the frame.

In contrast with the good LCA results, the acoustic simulation showed that the use of the bio-composites significantly decreases the airborne acoustic insulation. The sound reduction index drops from more than 42 decibels to around 31. This is probably due to the small thickness and lightweight of the boards since weight is one of the key elements of airborne sound isolation. Since the boards were designed with a sustainability goal in mind, their thickness was reduced to the maximum to decrease the amount of material while maintaining adequate mechanical properties. Decreasing the material amount used not only lowers the environmental impacts during production but also lowers the amount of waste generated at the end of life of the building. Despite the noticeable difference between the results, the airborne insulation is over 30 dBA in every bio-epoxy typology. This value has been accepted in most international legislations for many years if the wall divides rooms of the same housing unit. However, building acoustics legislations are getting more and more restrictive nowadays. It would be necessary to increase the thickness of the boards to provide enough airborne noise insulation, especially in countries with restrictive noise regulations. On the other hand, the influence that changing the acoustic absorbent has on the acoustic insulation of the studied partitions is almost negligible. This could be considered a good result because the inner acoustic absorbent could be replaced with one of the two alternatives presented, reducing the environmental impact, and not compromising the acoustic insulation.

## 5. Conclusions

The idea behind this project was to develop sustainable alternatives to conventional drywall partitions with mineral wool. As an alternative to plasterboard, a bio-composite board using Supersap resin and flax fiber was developed and manufactured in the university facilities. As for the inner layer, two materials were analyzed as alternatives to mineral wool. The first one is manufactured using recycled cellulose fiber, and the second one is produced with waste generated in the sheep wool industry. Six different partition configurations were studied combining the conventional and the bio-based materials mentioned. The environmental impacts of the partitions are compared by performing a life cycle assessment. A mathematical simulation was used to evaluate their airborne acoustic insulation. The main objective of the study was to determine the influence each element has over the environmental impacts and the acoustic insulation of the whole system. Therefore, it can be determined how much it is possible to lower the environmental impact while maintaining adequate acoustic insulation.

After finishing this study, several conclusions can be drawn. First, it can be stated that the bio-composite panels reduce the CO_2_ emissions of the whole partition by 50%. According to the results obtained using the ReCiPe endpoint method, the panels reduce the overall environmental impacts around 35%. Therefore, it can be said that the bio-composite panels analyzed significantly reduce the environmental impact when compared to plasterboard. In the case of the acoustic absorbers studied, using cellulose fiber reduces the total carbon emissions of the typology by 4.7% and 6.35% using sheep wool. The same results are obtained using the ReCiPe endpoint method, a reduction of 4.7% with cellulose fiber, and 6.35% with sheep wool. Although this difference is small, it would translate into a big reduction in the emissions considering the significant amount of materials required. The panels, however, have proved to lower the acoustic insulation while the acoustic absorbers offer a very similar result. The typology using the bio-composite panels and the sheep wool is the one that reduces the environmental impacts the most but also has the worst acoustic performance. Therefore, it can be concluded that using the bio-composite panels either with cellulose or sheep wool absorbent can be a sustainable option in cases where there is not a need for high airborne insulation. Consequently, it is necessary to improve the acoustic insulation of the partitions that use the bio-composite boards to comply with most acoustic legislations worldwide. Augmenting the amount of bio-epoxy resin to increase the weight would compromise the sustainability of the partition wall. Finding the optimum balance between both sustainability and acoustic insulation could be an interesting middle ground. It would be worth exploring other possibilities, such as using layers of different bio-based materials combined with the composite boards.

## Figures and Tables

**Table 1:** Materials in 1 m^2^ of typology.

Typology	Components	Mass (kg)	Total Mass (kg)
Plasterboard/MW	Plasterboard	12.0	16.9
	Mineral wool	3.5	
	Steel	1.40	
Plasterboard/Cellulose	Plasterboard	12.0	15.8
	Cellulose	2.4	
	Steel	1.40	
Plasterboard/Sheep wool	Plasterboard	12.0	14.9
	Sheep wool	1.5	
	Steel	1.4	
Bio-composite/MW	Bio-composite	5.1	9.9
	Mineral wool	3.5	
	Steel	1.4	
Bio-composite/Cellulose	Bio-composite	5.1	8.9
	Cellulose	2.4	
	Steel	1.4	
Bio-composite/Sheep wool	Bio-composite	5.1	7.9
	Sheep wool	1.5	
	Steel	1.4	

**Table 2:** Composite content proportions and mass per m^2^.

	Flax Board
Mass (kg)	5.08
Fiber %	49
Fiber mass (kg)	2.49
Bio-epoxy %	51
Bio-epoxy mass (kg)	2.59

**Table 3:** Physical and Mechanical Characteristics.

Board	Thickness (mm)	Density (g/cm^3^)	Mass/m^2^	Shore C Hardness ^1^	Impact Resistance ^2^
Plasterboard	12.5	0.776	9.7	47.7	14.9
Flax board	4.6	1.183	5.1	76.2	76.7

^1^ Shore Durometer model Instruments J.Bot 673D (ISO 868:2003). Scale Shore D 30°, ^2^ Charpy impact test. Pendulum by Metrotec (ISO 179:1993). Scale used: 1J.

**Table 4:** Data for the acoustic simulation.

**Air** **^1^**				
t, temperature (°C)			20	
c, sound velocity (m/s)			343	
ρ, density (kg/m^3^)			1.21	
**Boards** **^2^**				
	Bio-composite		Plasterboard	
m, mass (kg/m^2^)	5.51		13.5	
f_c_, frequency (kHz)	18.2		2.8	
η, loss factor	0.32		0.035	
**Acoustic Absorbents** **^3^**				
	Mineral wool	Cellulose		Sheep wool
σ, flux resistivity (rayls/m)	30,000	43,000		8300

^1^ Parameters found in [19]. ^2^ Measured following the methodology described in [20]. ^3^ Tested using an impedance tube following the methodology proposed in [21].

**Table 5:** Inventory of 1 kg of the sheep wool acoustic absorbent.

	Quantity	Unit
Sheep wool waste	0.85	kg
Polyethylene terephthalate (PET)	0.15	kg
Electricity medium voltage (ES)	1.588	MJ

**Table 6:** Inventory of 1 kg of cellulose fiber acoustic absorbent.

	Quantity	Unit
Wastepaper	1	kg
Boric acid, anhydrous, powder	0.111	kg
Borax, anhydrous, powder	0.136	kg
Electricity medium voltage	0.52	MJ
Heat, district, or industrial, natural gas	2.22	MJ

**Table 7:** Sound reduction index of each typology.

	R (dB)
Plasterboard/MW	42.7
Plasterboard/Cellulose	42.9
Plasterboard/Sheep wool	42.8
Composite/MW	32.0
Composite/Cellulose	31.9
Composite/Sheep wool	30.6

**Table 8:** Carbon dioxide emitted by each material in the partitions.

	Amount	Kg of CO_2_ Eq.
Plasterboard 1.5 cm	2 m^2^	57.87
Bio-composite 0.5 cm	2 m^2^	21.65
Galvanized steel frame	1.4 kg	7.64
Mineral wool	3.5 kg	4.89
Cellulose fiber	2.4 kg	1.45
Sheep wool	1.5 kg	0.74

**Table 9:** ReCiPe midpoint characterization.

Impact Category	Unit	Plast./MW	Plast./Cell.	Plast./Sheep Wool	Comp./MW	Comp./Cell.	Comp./Sheep Wool
Climate change	kg CO_2_ eq.	69.7412	66.3669	65.6729	33.4817	30.1074	29.4135
Ozone depletion	kg CFC-11 eq.	0.0000	0.0000	0.0000	0.0000	0.0000	0.0000
Terrestrial acidification	kg SO2 eq.	0.2236	0.2013	0.1947	0.2026	0.1804	0.1737
Freshwater eutrophication	kg P eq.	0.0095	0.0084	0.0081	0.0098	0.0087	0.0083
Marine eutrophication	kg N eq.	0.0115	0.0110	0.0105	0.0082	0.0077	0.0073
Human toxicity	kg 1,4-DB eq.	18.6697	17.6842	17.0793	19.2523	18.2669	17.6619
Photochemical oxidant formation	kg NMVOC	0.1841	0.1662	0.1619	0.1916	0.1736	0.1694
Particulate matter formation	kg PM10 eq.	0.0867	0.0799	0.0752	0.1060	0.0991	0.0945
Terrestrial ecotoxicity	kg 1,4-DB eq.	0.0064	0.0065	0.0061	0.0049	0.0050	0.0046
Freshwater ecotoxicity	kg 1,4-DB eq.	0.3562	0.3491	0.3313	0.4474	0.4402	0.4225
Marine ecotoxicity	kg 1,4-DB eq.	0.3972	0.3906	0.3735	0.4649	0.4583	0.4412
Ionizing radiation	kBq U235 eq.	4.1787	4.2771	4.1624	0.5287	0.6270	0.5123
Agricultural land occupation	m^2^a	0.9723	1.8088	0.7931	1.6142	2.4506	1.4349
Urban land occupation	m^2^a	0.3818	0.3616	0.3162	0.2466	0.2263	0.1809
Natural land transformation	m^2^	0.0053	0.0038	0.0034	0.0033	0.0018	0.0014
Water depletion	m^3^	0.4771	0.4696	0.4622	0.5845	0.5770	0.5696
Metal depletion	kg Fe eq.	6.5388	6.3924	6.2534	6.4010	6.2546	6.1156
Fossil depletion	kg oil eq.	21.2216	20.1604	20.1179	11.6111	10.5500	10.5074
